# CT-Based Finite-Element Analysis of the Optimal Olecranon Osteotomy Angle for Tension Band Wiring

**DOI:** 10.7759/cureus.87101

**Published:** 2025-07-01

**Authors:** Takahiro Yamazaki, Akitoshi Sakuma, Yusuke Matsuura, Takane Suzuki, Takuto Takeda, Seiji Ohtori

**Affiliations:** 1 Department of Orthopedic Surgery, Chiba University, Chiba, JPN

**Keywords:** contact pressure distribution, finite-element method, olecranon osteotomy, osteotomy angle, tension band wiring technique

## Abstract

Background: Olecranon osteotomy is commonly used for surgical exposure in distal humeral fractures, with subsequent fixation typically performed using tension band wiring (TBW). However, the optimal osteotomy angle for maximizing interfragmentary compression remains unclear.

Methods: Using finite-element analysis of a 30-year-old male's elbow CT data, we analyzed seven osteotomy angles relative to the ulnar axis: 20° proximal (P20), 10° proximal (P10), vertical (V), 10° distal (D10), 20° distal (D20), 30° distal (D30), and 40° distal (D40). Wire tightening force (30N) and triceps traction force (100 N) were applied sequentially. Contact pressure was measured across the entire osteotomy surface and the articular half.

Results: The 20° distal angle (D20) produced maximum contact pressure (317 kPa) across the entire osteotomy surface compared to other angles (P20: 237 kPa, P10: 295 kPa, V: 276 kPa, D10: 259 kPa, D30: 171 kPa, D40: 130 kPa). Articular-side pressure was also highest at D20 (206 kPa). The ratio of articular-side to total pressure increased progressively with more distal angles, from 23% (P20) to 80% (D40).

Conclusions: A 20° distal osteotomy angle provides optimal biomechanical conditions for TBW fixation, maximizing interfragmentary compression essential for bone healing. This finding may guide surgical technique selection to improve clinical outcomes.

## Introduction

Distal humeral fractures represent a challenging clinical entity, occurring at 5.7 cases per 100,000 people and comprising approximately 2% of all fractures [1,2]. Intra-articular involvement occurs in 61% of cases, presenting significant technical difficulties due to the complex articular geometry of the capitellum and trochlea [1].

Since Cassebaum's initial description in 1952, olecranon osteotomy has become a standard approach for adequate surgical exposure in complex distal humeral fractures [3]. This technique provides excellent visualization of the articular surface, facilitating anatomical reduction and stable internal fixation. Following fracture repair, the osteotomized olecranon requires secure fixation to restore elbow function and prevent complications.

Various fixation methods have been described for olecranon osteotomy repair, including screw fixation, tension band wiring (TBW), and plate fixation [3-5]. Despite extensive clinical experience, no consensus exists regarding the optimal technique. TBW remains widely utilized due to its technical simplicity and effectiveness in converting tensile forces into compressive forces across the osteotomy site [6].

The biomechanical principle underlying TBW involves transforming distractive forces that would separate bone fragments into compressive forces that promote healing [6]. Adequate interfragmentary compression generates frictional forces at the contact interface, enhancing stability, minimizing micromotion, and facilitating direct bone union. This compression is particularly critical near the articular surface, where stability directly impacts joint congruity and functional outcomes.

Previous investigations have examined fragment displacement patterns with varying osteotomy angles [7,8], but none have directly quantified contact pressure distribution across different osteotomy orientations. Understanding the relationship between osteotomy angle and interfragmentary pressure distribution could optimize surgical technique and improve clinical outcomes.

Finite-element analysis (FEA) has emerged as a powerful tool for biomechanical investigation in orthopedic surgery. CT-based FEA enables the creation of patient-specific models with validated accuracy against cadaveric specimens [9]. These methods have proven valuable for analyzing complex mechanical behaviors within bone structures that cannot be directly measured clinically.

We hypothesized that interfragmentary compressive force distribution in TBW fixation varies significantly with osteotomy angle, and that an optimal angle exists for maximizing contact pressure. This study aimed to determine the optimal olecranon osteotomy angle using FEA by measuring contact pressure distribution across various osteotomy orientations.

## Materials and methods

CT imaging and 3D model creation

CT imaging was performed on a 30-year-old healthy male volunteer with no history of upper extremity fractures or bone diseases. Imaging was conducted using an Aquilion ONE scanner (Toshiba Medical Systems, Tokyo, Japan) with the following parameters: 320-row detector, 120 kV, 200 mA, pixel width 0.3 mm, and slice thickness 0.5 mm.

Digital Imaging and Communications in Medicine data were imported into an HP Z8 workstation (Hewlett-Packard Company, California, USA) and processed using Mechanical Finder version 12 finite-element analysis software (Research Center for Computational Mechanics, Tokyo, Japan). The region of interest was extracted to create a 3D model with the elbow positioned at 90° flexion. Hardware components (2.0-mm Kirschner wire and 1.0-mm soft steel wire) were modeled using the Metasequoia 4 software (Tetraface Inc., Tokyo, Japan).

Osteotomy design

**Figure 1:**
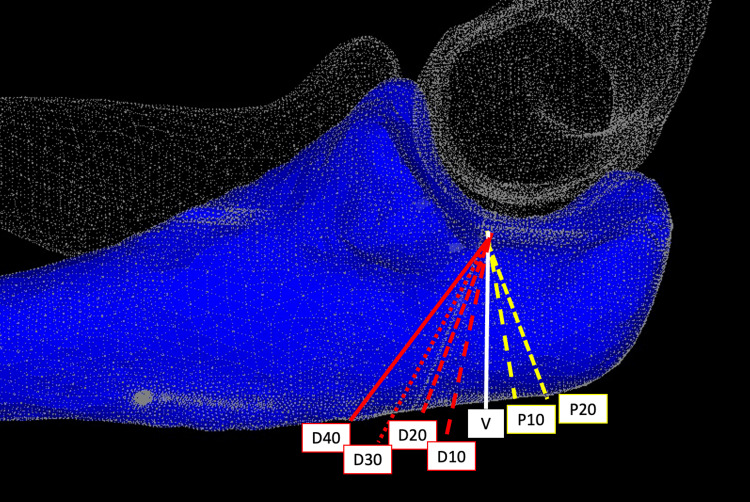
Models for each osteotomy angle Models were created with osteotomy angles of 10° proximal (P10), 20° proximal (P20), vertical (V), 10° distal (D10), 20° distal (D20), 30° distal (D30), and 40° distal (D40) relative to the ulnar axis Image credit: This is an original image created by the author Akitoshi Sakuma

The osteotomy line was positioned to traverse the "bare area" described by Morrey to replicate clinical practice [10]. Seven osteotomy angles were created relative to the ulnar axis: 20° proximal (P20), 10° proximal (P10), vertical (V), 10° distal (D10), 20° distal (D20), 30° distal (D30), and 40° distal (D40) (Figure 1).

The soft steel wire insertion site in the ulnar shaft was positioned at a distance equal to four times the trochlear radius, based on previously reported optimal positioning [11].

Finite-element model development

Mesh Generation

The 3D model utilized tetrahedral elements of 2-3 mm for the main bone structure, with cortical bone surfaces reproduced using 2-3 mm triangular shell elements. Hardware components (K-wire and soft steel wire) employed finer 0.2-0.3 mm tetrahedral elements. The osteotomy region, where contact pressure was evaluated, utilized refined 1-1.5 mm tetrahedral elements for enhanced accuracy. Articular cartilage between the humerus, radius, and ulna was modeled using 2-3 mm tetrahedral elements.

Material Properties

Bone material properties were assigned based on CT Hounsfield unit values using validated conversion equations [12]:

Young's modulus (E): E = 1530.6 × ρ^1.9213^ (MPa)

Yield stress: σ = 116.64 × ρ^1.8952^ (MPa)

Hardware components utilized stainless steel properties (SUS304): Young's modulus 196,133 MPa and Poisson's ratio 0.34. Articular cartilage properties were set as: Young's modulus 10 MPa, Poisson's ratio 0.45 [13]. Critical stress and yield stress values were set sufficiently high to prevent failure during calculation.

Boundary and Loading Conditions

**Figure 2:**
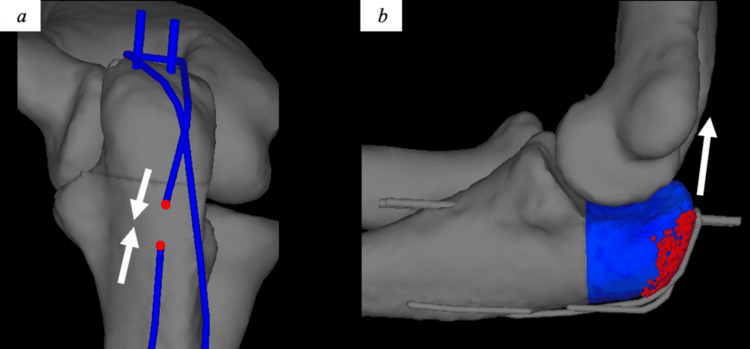
Tension band model created based on 3D CT (a) Blue represents the 2-mm K-wire and 1-mm soft steel wire. The end of the soft steel wire shown in red was pulled in the direction of the arrow with a force of 30 N and maintained. (b) Blue represents the olecranon fragment, and the red dot indicates the triceps brachii attachment. After tightening the soft steel wire, the olecranon fragment was pulled in the direction of the white arrow 3D: three dimensional Image credit: This is an original image created by the author Akitoshi Sakuma

Contact conditions were established between all interacting surfaces (bone fragments, cartilage, and hardware) with a friction coefficient of 0 to isolate pure contact pressure effects. Boundary conditions were applied to simulate physiological constraints during the surgical procedure. The proximal humerus was fixed in all degrees of freedom to represent stable proximal fixation, while the distal radius and ulna were constrained to prevent rigid body motion while allowing natural joint mechanics. Loading was applied in two sequential phases to simulate clinical conditions: 1) wire tightening phase: gradual application of 2 N increments to the soft steel wire up to 30 N; and 2) triceps traction phase: progressive application of 2 N increments to the olecranon (at the triceps insertion) up to 100 N, based on physiological triceps tension at 90° elbow flexion [14]. The soft steel wire tension was maintained at 30 N throughout the triceps loading phase using displacement control (Figure 2).

Outcome Measurements

Contact pressure (kPa) was calculated by dividing the total contact reaction force by the corresponding surface area. Measurements excluded the K-wire passage region to eliminate local stress concentrations from hardware insertion. The primary outcomes included contact pressure across the entire osteotomy surface, contact pressure on the articular half of the osteotomy surface, and the ratio of articular-half pressure to total surface pressure. All measurements were recorded at maximum triceps loading (100 N) for comparison of osteotomy angles.

## Results

**Figure 3:**
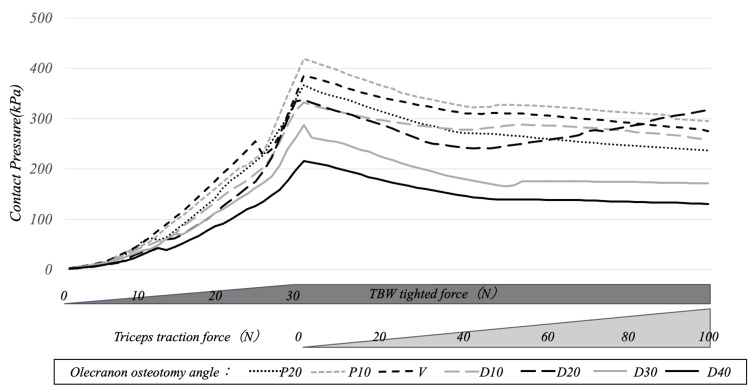
Transition of contact pressure on the entire osteotomy surface during tension band tightening and olecranon traction The contact pressure on the entire osteotomy surface increased with each increase in soft steel wire tightening and gradually decreased with the traction of the olecranon by the triceps brachii. By osteotomy angle, only in D20 did the contact pressure gradually increase from the point at which the olecranon traction force exceeded 50 N TBW: tension band wiring

**Figure 4:**
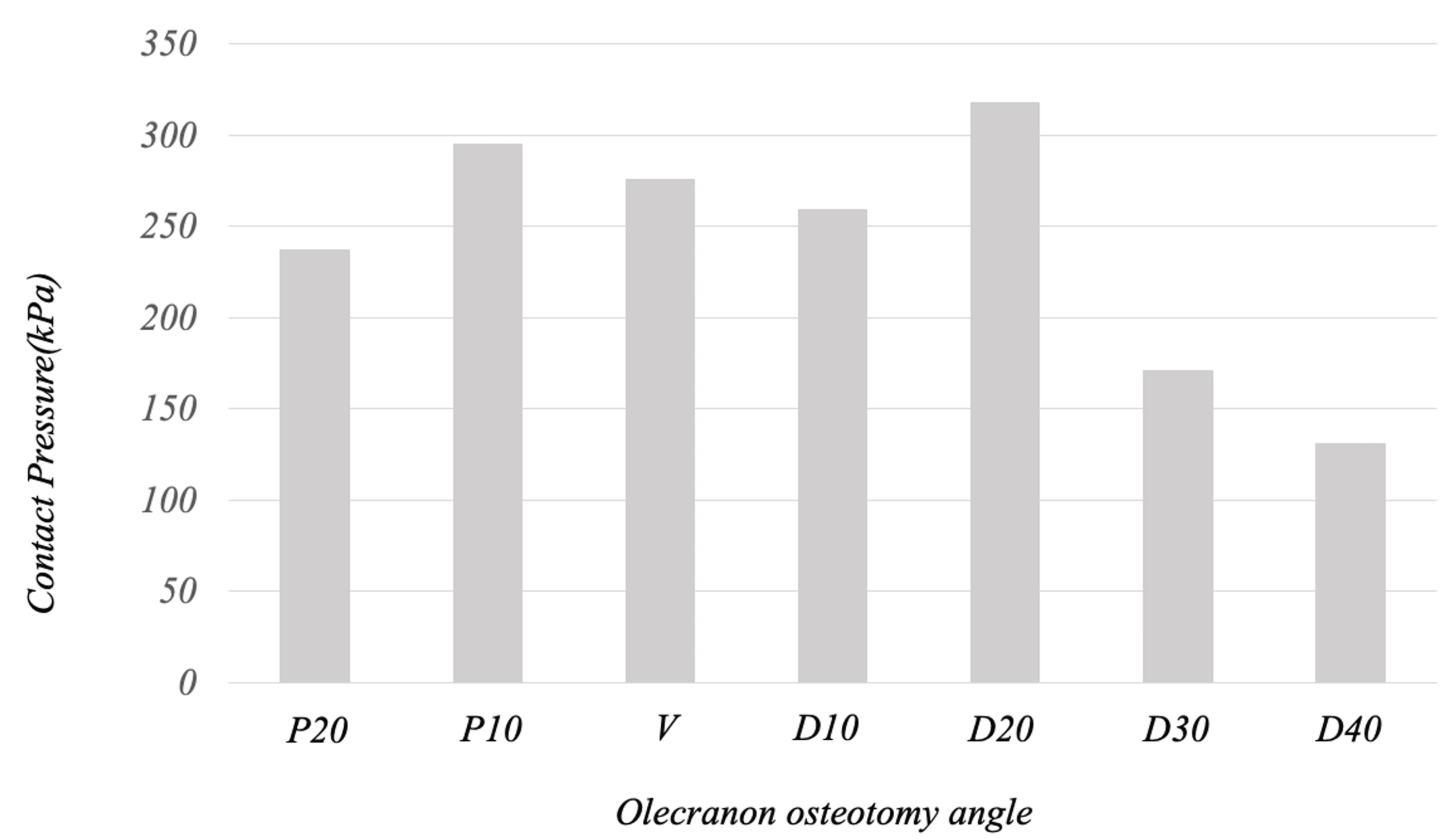
Contact pressure on the entire osteotomy surface at the end of olecranon traction At the end of olecranon traction, the contact pressure on the entire osteotomy surface was 237, 295, 276, 259, 317, 171, and 130 kPa for P20, P10, V, D10, D20, D30, and D40, respectively, with the D20 model showing the maximum value

The contact pressure on the entire osteotomy surface increased as the soft steel wire tightening was strengthened and gradually decreased with the traction of the olecranon by the triceps brachii. By osteotomy angle, only in D20 did the contact pressure gradually increase from the point at which the olecranon traction force exceeded 50 N. At the end of olecranon traction, the contact pressure on the entire osteotomy surface was 237, 295, 276, 259, 317, 171, and 130 for P20, P10, V, D10, D20, D30, and D40, respectively, with the D20 model showing the maximum value (Figures 3, 4).

**Figure 5:**
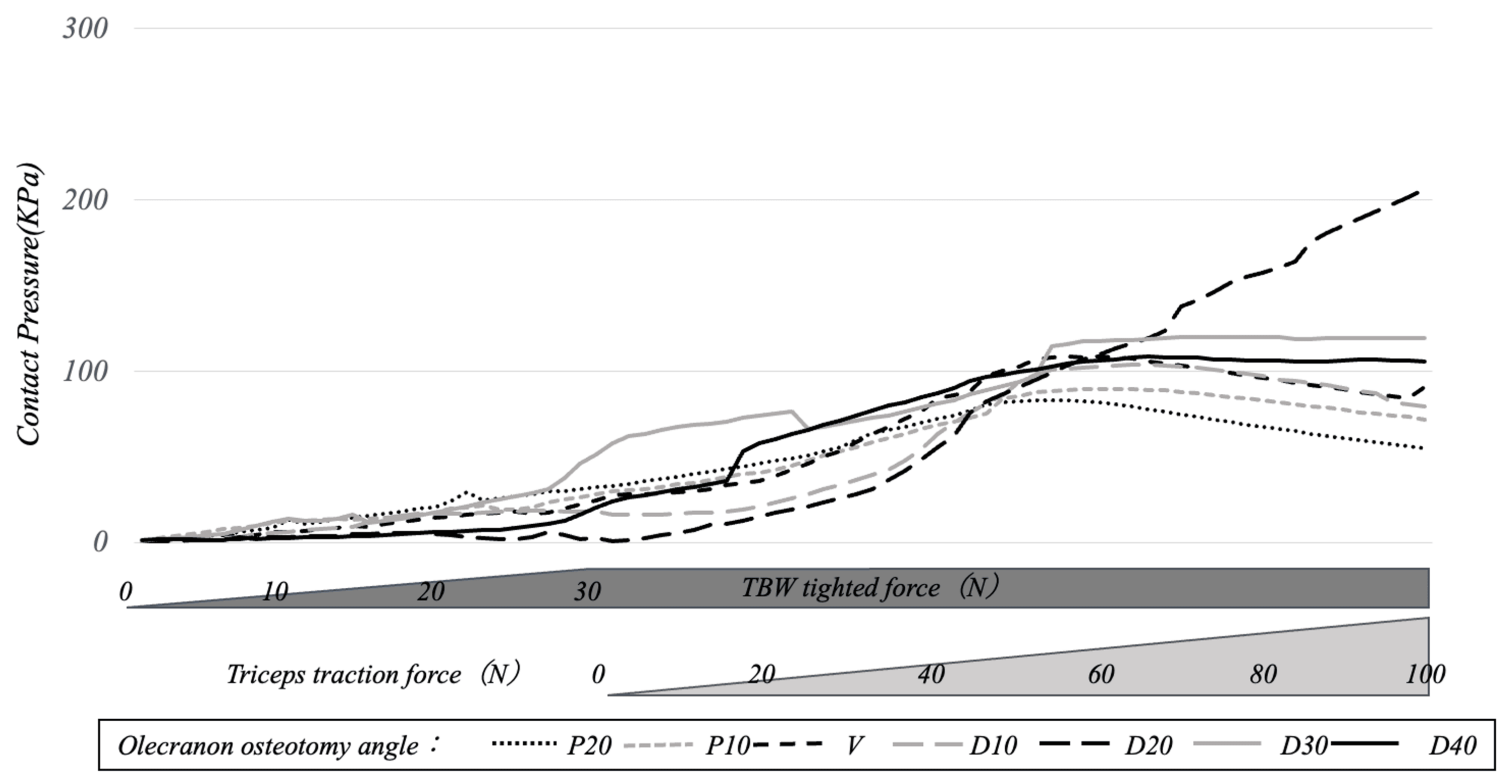
Transition of contact pressure on the articular side 1/2 of the osteotomy surface during tension band tightening and olecranon traction The contact pressure on the articular side 1/2 gradually increased from the soft steel wire tightening, reaching a maximum at the point when 50-60 N of olecranon traction force was applied in P20, P10, V, and D10, then gradually decreased. In D20, the contact pressure continued to increase until 100 N of olecranon traction, while in D30 and D40, it plateaued at 50-60 N of olecranon traction TBW: tension band wiring

**Figure 6:**
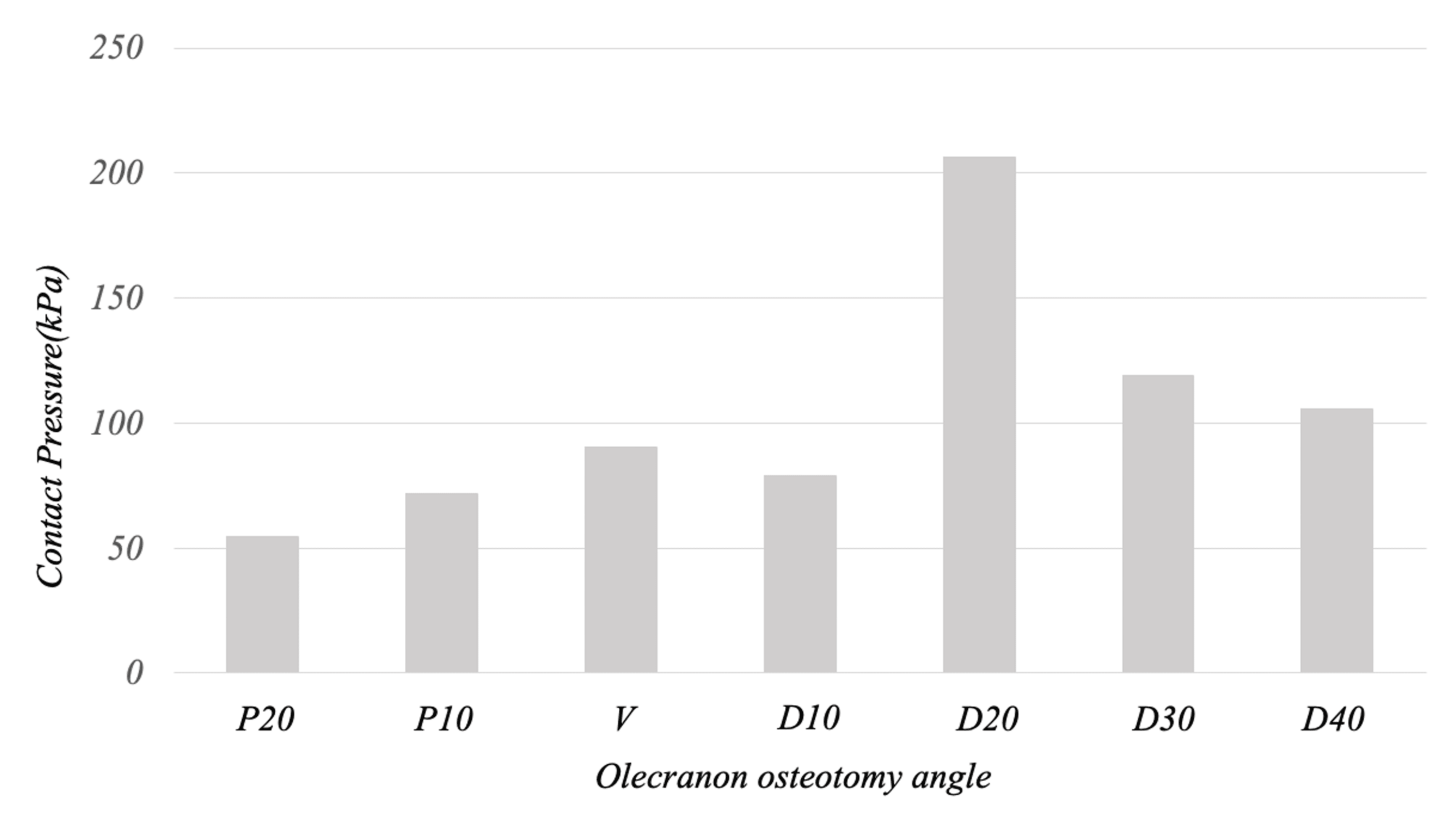
Contact pressure on the articular side 1/2 of the osteotomy surface at the end of olecranon traction At the end of olecranon traction, the contact pressure on the articular side 1/2 was 55, 72, 91, 79, 206, 119, and 106 kPa for P20, P10, V, D10, D20, D30, and D40, respectively, with D20 showing the maximum value

The contact pressure on the articular side 1/2 gradually increased from the soft steel wire tightening, reaching a maximum at the point when 50-60 N of olecranon traction force was applied in P20, P10, V, and D10, then gradually decreased. In D20, the contact pressure continued to increase until 100 N of olecranon traction, while in D30 and D40, it plateaued at 50-60 N of olecranon traction. At the end of olecranon traction, the contact pressure on the articular side 1/2 was 55, 72, 91, 79, 206, 119, and 106 kPa for P20, P10, V, D10, D20, D30, and D40, respectively, with D20 showing the maximum value (Figures 5, 6).

**Figure 7:**
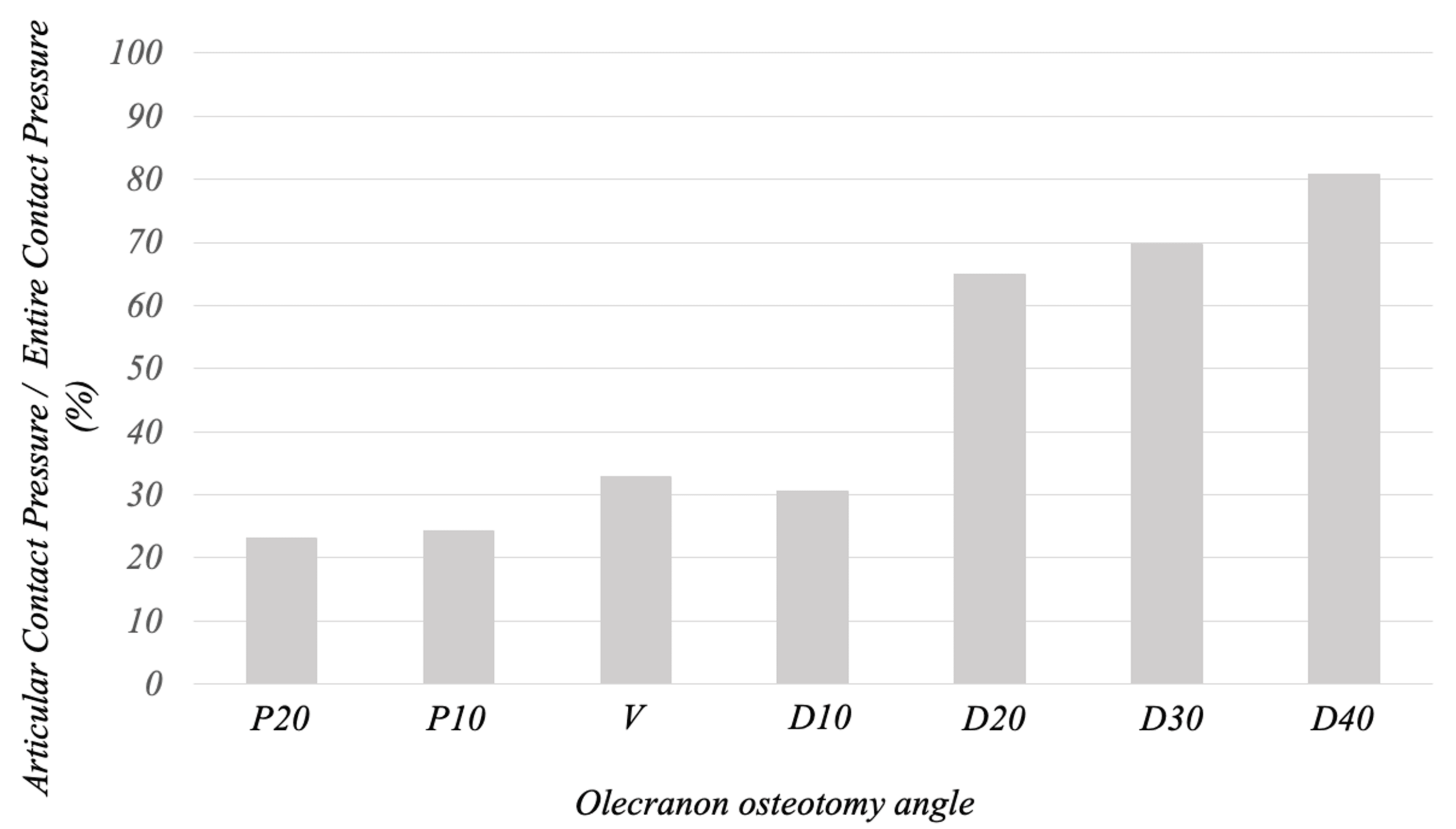
Ratio of contact pressure on the articular side 1/2 to the entire osteotomy surface at the end of olecranon traction At the end of olecranon traction, the ratio of the contact pressure on the articular side 1/2 to that on the entire osteotomy surface was 23%, 24%, 33%, 31%, 65%, 70%, and 80% for P20, P10, V, D10, D20, D30, and D40, respectively, indicating that the contact pressure was more widely distributed to the articular side 1/2 as the osteotomy angle became more distal

At the end of olecranon traction, the ratio of the contact pressure on the articular side 1/2 to that on the entire osteotomy surface was 23%, 24%, 33%, 31%, 65%, 70%, and 80% for P20, P10, V, D10, D20, D30, and D40, respectively, indicating that the contact pressure was more widely distributed to the articular side 1/2 as the osteotomy angle became more distal (Figure 7).

## Discussion

Principal findings

This FEA demonstrates that osteotomy angle significantly influences interfragmentary contact pressure distribution in TBW fixation. The 20° distal angle (D20) produced optimal biomechanical conditions, maximizing both total and articular side contact pressure while maintaining favorable pressure distribution.

Biomechanical mechanisms

**Figure 8:**
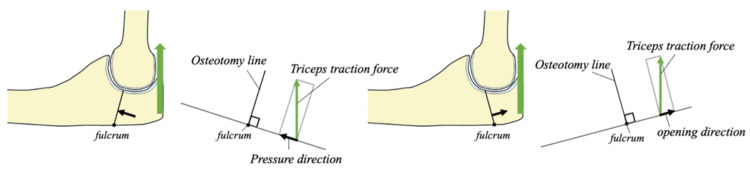
Schematic diagram and force relationship of D20 and P20 During olecranon traction, the olecranon fragment displaces with the posterior cortical bone of the osteotomy site as a fulcrum. In D20, the olecranon traction causes the fragment to displace in the compression direction, while in P20, the olecranon fragment tends to displace in the separation direction due to traction Image credit: This is an original image created by the author Akitoshi Sakuma

The superior performance of the D20 configuration can be explained by force vector analysis. During triceps contraction, the olecranon fragment rotates about the posterior cortical contact point established by wire tension. In proximal-angled osteotomies (P20, P10), triceps force vectors decompose partially in the direction of fragment separation, opposing wire tension and reducing interfragmentary pressure (Figure 8).

Conversely, distal-angled osteotomies redirect triceps force vectors toward fragment compression, augmenting wire-generated pressure. The D20 angle appears to represent the optimal balance where force vectors act most efficiently perpendicular to the osteotomy surface, maximizing compressive force conversion.

Beyond D20, excessive angulation (D30, D40) may increase fragment interlocking but reduce the perpendicular force component, diminishing overall contact pressure despite favorable pressure distribution ratios.

Clinical implications

These findings align with established fracture healing principles outlined in Perren's interfragmentary strain theory [15]. Adequate interfragmentary compression reduces micromotion, promoting direct bone healing rather than callus formation. The 65% articular side pressure distribution achieved with D20 is particularly significant, as articular surface stability directly impacts joint congruity and enables early mobilization.

Current clinical practice often defaults to vertical or slightly oblique osteotomies based on surgeon preference rather than biomechanical optimization [7]. Our biomechanical findings indicate that a 20° distal angle produces optimal contact pressure distribution in this computational model. However, clinical validation through prospective studies is necessary to confirm whether these biomechanical advantages translate to improved union rates and clinical outcomes.

Comparison with previous studies

Previous mechanical studies have produced conflicting results regarding optimal osteotomy angles. Petraco et al. found no significant differences in stability between chevron, vertical, and oblique osteotomies using cadaveric testing [7]. Similarly, Neat et al. reported equivalent performance between vertical and 15° distal oblique osteotomies in acetyl resin models [8].

These apparent contradictions likely reflect methodological differences. Previous studies measured fragment displacement under loading, which may not capture the subtle pressure distribution differences revealed by FEA. Our approach directly quantifies interfragmentary pressure, providing more sensitive detection of biomechanical advantages.

Study limitations

Several limitations warrant consideration in interpreting these results. Single-subject modeling presents the first limitation, as this analysis utilized CT data from one healthy 30-year-old man, potentially limiting generalizability across different patient populations and bone geometries. Simplified material properties constitute another consideration, particularly the friction-free contact assumption, which, while eliminating confounding variables, may not fully represent in vivo conditions where friction contributes to stability. Static loading conditions represent a third limitation, as real-world loading involves dynamic, multidirectional forces that may produce different pressure distributions. Age considerations also warrant attention, as the young, healthy bone model may not reflect the typical patient population requiring olecranon osteotomy, who often present with age-related bone quality changes. Finally, clinical validation remains necessary, as the relationship between computed contact pressure and actual bone union rates requires verification through clinical studies.

Future directions

These computational findings provide a foundation for clinical investigation. Prospective studies comparing union rates, complication rates, and functional outcomes between different osteotomy angles would validate the clinical relevance of these biomechanical advantages. Additionally, expanding the FEA to include multiple patient models would strengthen the generalizability of results.

Investigation of dynamic loading conditions and the inclusion of physiological friction coefficients would enhance model realism. Long-term studies examining the relationship between initial fixation stability and ultimate clinical outcomes would provide valuable insights into the clinical significance of optimized osteotomy angles.

## Conclusions

This FEA demonstrates that osteotomy angle significantly influences interfragmentary contact pressure in TBW fixation of olecranon osteotomies. A 20° distal angle relative to the ulnar axis provides optimal biomechanical conditions, maximizing both total interfragmentary pressure (317 kPa) and articular-side pressure (206 kPa) while achieving favorable pressure distribution (65% articular-side).

These findings suggest that systematic adoption of a 20° distal osteotomy angle may enhance fixation stability and promote bone union in patients requiring olecranon osteotomy for distal humeral fracture exposure. Clinical validation through prospective studies is warranted to confirm the practical significance of these biomechanical advantages.
